# Quantifying the Domino Effect of Septoplasty on Eustachian Tube Function and Nasal Resistance in Patients With Chronic Otitis Media: A Cross-Sectional Study

**DOI:** 10.7759/cureus.69819

**Published:** 2024-09-20

**Authors:** Nimisha Patil, Shraddha Jain, Prasad T Deshmukh, Sagar S Gaurkar, Chandra Veer Singh, Megha Kawale, Smriti Wadhwa, Jaya Gupta, Abhijeet Sharma, Parindita Sarmah

**Affiliations:** 1 Department of Otolaryngology - Head and Neck Surgery, Jawaharlal Nehru Medical College, Datta Meghe Institute of Higher Education and Research (DMIHER), Wardha, IND; 2 Department of Otorhinolaryngology - Head and Neck Surgery, Jawaharlal Nehru Medical College, Datta Meghe Institute of Higher Education and Research (DMIHER), Wardha, IND

**Keywords:** chronic otitis media, deviated nasal septum, dynamic slow video endoscopy, eustachian tube function, nasal resistance, rhinomanometry

## Abstract

Background

Eustachian tube dysfunction is characterized by insufficient dilation, leading to secondary pathologies in the middle ear. By comparing pre- and post-operative grades of Eustachian tube function and nasal resistance measurements, this study seeks to determine if septoplasty can improve Eustachian tube function in cases where nasal septal deviation is likely to cause mechanical dysfunction. We also aim to validate the Jain Bhalerao endoscopic classification of nasal septal deviation by assessing its utility in identifying septal deviations at a higher risk of causing Eustachian tube dysfunction. The ultimate goal is to establish guidelines for aural indications of septoplasty in treating Eustachian tube dysfunction-related middle ear disorders.

Material and methods

This prospective observational study was carried out from 1^st^ June 2022 to 31^st^ March 2024 in the Department of Otorhinolaryngology at Acharya Vinoba Bhave Rural Hospital, involving 66 patients diagnosed with chronic otitis media and a deviated nasal septum with Eustachian tube dysfunction. Pre-operative and post-operative improvement in Eustachian tube function and Nasal resistance and their correlation were studied using dynamic slow-motion video endoscopy and active anterior rhinomanometry, respectively. Statistical analysis included the chi-square test.

Results

Sixty-six patients diagnosed with chronic otitis media and deviated nasal septum with Eustachian tube dysfunction included in this study had a mean age of 35.6 years with 43 (65.2%) male predominance. Gross luminal narrowing and discrepancy of the volume of both nasal cavities led to higher degrees of Eustachian tube dysfunction due to pressure drop on the affected side, as observed in types 6, 7, and 8 as per Jain Bhalerao classification of deviated nasal septum. Nasal resistance measured using active anterior Rhinomanometry (Rhinodebitometry) showed a positive association of severity of nasal resistance in the types 6, 7, and 8 DNS of the Jain Bhalerao classification of deviated nasal septum. It demonstrated an improvement in the grade of Eustachian tube function and nasal resistance post-septoplasty.

Conclusion

The deviated nasal septum is one of the causes of Eustachian tube dysfunction and increased nasal resistance. Certain types of DNS are adversely associated with the causation of greater degrees of Eustachian tube dysfunction of mechanical type. Nasal septal deviation correction improved Eustachian dysfunction and nasal resistance after septoplasty.

## Introduction

The Eustachian tube dysfunction is characterized by insufficient dilatory function leading to secondary ear pathology [1]. The spectrum of middle ear pathologies secondary to Eustachian dysfunction comprises high negative pressure in the middle ear, causing atelectasis of the tympanic membrane, resulting in the formation of a retraction pocket in either the posterosuperior or attic portion of the tympanic membrane, which progresses to adhesive otitis media or acquired cholesteatoma [2,3]. Eustachian tube dysfunction is classified into mechanical and functional dysfunction [4]. The functional dysfunction is due to the inherent weakness of tubal muscles. The mechanical type of tubal dysfunction is characterized by intraluminal edema and reduced muscle mobility due to nasal, paranasal, and nasopharyngeal etiologies [4]. It is attributable to conditions of infective, allergic, or obstructive nature in the nose, nasopharynx, or paranasal sinuses [5]. This leads to various otologic conditions due to inadequate dilatory function. The role of the deviated nasal septum in the causation of Eustachian tube dysfunction is controversial. Few studies have correlated deviated nasal septum with Eustachian tube dysfunction and the effect of nasal surgeries in patients with chronic otitis media [6-8]. Although some researchers have supported that nasal septal deviation (NSD) could worsen middle ear ventilation by negative influence on airflow parameters and advocate that severe nasal septal deviation can be the cause of failure of ear surgery for chronic otitis media, other studies failed to prove the association [8-10]. There is a lot of contention regarding the role of septal correction in middle ear pathologies [11]. This is due to a lack of an objective standard classification of deviated nasal septum (DNS). Jain Bhalerao classification is an anatomic endoscopic classification of deviated nasal septum (DNS), which emphasizes fixed bony deviations causing luminal discrepancy as the culprit for the causation of Eustachian tube dysfunction [11]. Given the absence of a gold-standard method to evaluate Eustachian tube function, studies that address the impact of Nasal septal deviation on Eustachian tube dysfunction use different methods for its evaluation, which can also make their results difficult to interpret and compare [7-10]. Hence, assessing and correcting Eustachian tube dysfunction is paramount in treating middle ear disorders. Dynamic slow-motion video endoscopic (DSVE) analysis of the Eustachian tube helps in understanding the pathophysiology of tubal dysfunction and can help to classify into functional and mechanical causes by directly visualizing the nasopharyngeal opening of the Eustachian tube during rest, swallowing, and yawning [12]. Rhinomanometry (RMM) provides an objective assessment method for nasal airflow and resistance. Hence, it could be utilized as a tool for quantification of the degree of nasal resistance in different types of nasal septal deviations [13,14]. It would also show the impact of luminal discrepancy by objective assessments of nasal resistance on the causation and severity of Eustachian tube dysfunction. The use of rhinomanometry (RMM) as a pre-operative and post-operative assessment tool can help validate the utility of septoplasty surgeries in certain types of nasal septal deviations causing Eustachian tube dysfunction in patients with chronic otitis media [13,14]. With these considerations, the present study was undertaken. This study aims to validate objectively the Jain Bhalerao classification of nasal septal deviation for its utility in identifying the septal deviations at higher risk of Eustachian tube dysfunction by studying variations in nasal resistance (assessed by rhinodebitometry) [11]. Also, the Eustachian tube function should improve after septoplasty if the nasal septum was likely an etiology for mechanical dysfunction [11]. In order to test this hypothesis, the current study also aims to compare the pre- and post-operative grade of eustachian tube dysfunction with nasal resistance measured after septoplasty. This would help formulate the guidelines for aural indications of septoplasty.

## Materials and methods

This prospective observational study was carried out on 66 patients, in the Department of Otorhinolaryngology at Acharya Vinoba Bhave Rural Hospital, Sawangi (Meghe), Wardha, affiliated with Jawaharlal Nehru Medical College, Sawangi (Meghe), Wardha, Central India, from 1st June 2022 to 31st March 2024. The hospital primarily serves a rural population from East Maharashtra. Approval from the Institutional Ethics Committee was obtained for this study [Ref No. DMIHER (DU)/IEC/2023/1326].

**Figure 1:**
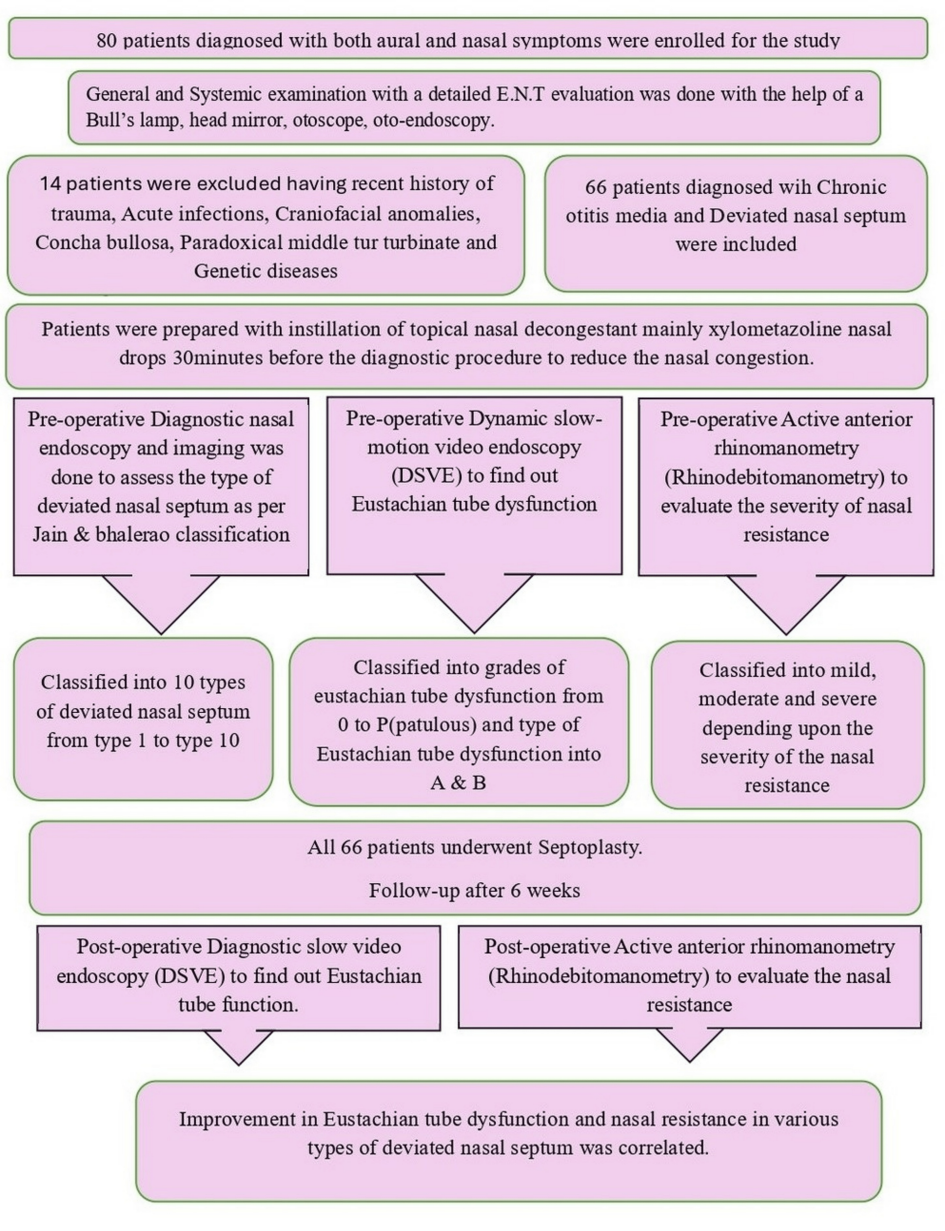
Methodology flowchart ETD: Eustachian tube dysfunction, DNS: Deviated nasal septum, DSVE: Dynamic slow-motion video endoscopy

The study initially enrolled 80 patients diagnosed with chronic otitis media (COM) who also had deviated nasal septum (DNS) and eustachian tube dysfunction (ETD). The study was undertaken to see the contribution of DNS in the causation of ETD in patients with COM. Chronic otitis media included both mucosal and squamous types, active and inactive. However, out of these 80 patients, 14 were excluded based on exclusion criteria as shown in Figure 1. Finally, 66 patients were included in the study to see the effect of different types of deviated nasal septum in the causation of Eustachian tube dysfunction in patients with COM [15]. Septal deviations were classified as per Jain Bhalerao's endoscopic anatomic classification, and each type's influence in causation and degree of Eustachian tube dysfunction was studied [11]. This classification, the only endoscopic anatomic classification with objective parameters for different types and not a geometric one, was chosen for the present study [11]. The ETD was studied with dynamic slow-motion video endoscopy (DSVE) [16]. Nasal resistance measurements were done by active anterior rhinomanometry (rhinodebitometry) for different types of DNS of Jain Bhalerao classification [11,17]. The ETD was correlated with nasal resistance findings to objectively assess the correlation of nasal resistance with ETD [17,18]. The above observations led us to conclude the type of DNS is more frequently associated with ETD. Septoplasty was performed in all 66 patients. Repeat investigations (ETD by DSVE, nasal resistance by active anterior rhinomanometry) were performed six weeks after surgery in the postoperative period to study the improvement in Eustachian dysfunction and nasal resistance as a benefit of surgery. The association between improvement in nasal resistance in patients with deviated nasal septum undergoing septoplasty and the degree of improvement in eustachian function was studied, and statistical tests were applied (Chi-square test).

Sample size

In research, a sample is a group of people, objects, or items taken from a larger population for measurement. The sample should be representative of the population to ensure that we can generalize the findings from the research sample to the population as a whole. Sample size formula with the designed error of margin: 

Daniel formula: n = Z^2^ .p.(1-p) )/d^2^

Z is the statistics for the level of significance at 95%, confidence interval = 1.96, P = prevalence of DNS = 20%, E = desired error of margin = 10% = 0.10, n = 1.96^2^ x 0.20 x (1 - 0.20)/ 0.10^2 ^= 61.46, n = 65 patients needed in the study.

Study design

Prospective cross-sectional study. So, the minimum sample size required will be 66 patients. 

Inclusion criteria

All the consecutive/sequential patients in the age group of 18 years to 70 years were diagnosed with chronic otitis media and deviated nasal septum with Eustachian tube dysfunction.

Exclusion criteria

History of recent trauma, neoplasms of the nose/PNS, acute infections, craniofacial anomalies, concha bullosa, paradoxical middle turbinate/double middle, patients below the age group of 18 years, lack of follow-up, DNS types mainly 1, 2, 9, and 10 of the Jain Bhalerao anatomic endoscopic classification could not be included as no participants were found of these types during the study period. Family history and congenital diseases like Downs syndrome and Treacher-Collins syndrome were excluded.

Statistical analysis

IBM Corp. Released 2016. IBM SPSS Statistics for Windows, Version 24.0. Armonk, NY: IBM Corp. was used to evaluate the data. The average score was computed and assessed. The correlation between Eustachian tube dysfunction and nasal resistance in the preoperative and postoperative periods was studied using a chi-square test. If the p-value was less than 0.05, the results were deemed statistically significant at a 95% confidence level.

## Results

Baseline characteristics and clinical features

Out of 66 patients enrolled in the study, the mean age was 35.6 ± 9.9 years, with a minimum age of 18 years and a maximum age of 66 years, of which 43 (65.2%) were males and 23 (34.8%) were females. All 66 patients complained of nasal obstruction, aural blockage, and reduced hearing, and 64 patients complained of ear discharge. All 66 patients were diagnosed with chronic otitis media (COM), out of which 44 were mucosal types and the rest were squamous types. All other baseline characteristics and clinical features are shown in Table 1.

**Table 1:** Clinical features COM: Chronic otitis media

Characteristics	No of Patients (n = 66)	Percentage (%)
Clinical features		
Nasal Obstruction	66	100%
Snoring	50	75.7%
Aural blockage	66	100%
Reduced Hearing	66	100%
Ear Discharge	64	96.9%
Ringing Sensation	26	39.39%
Type of COM (Chronic Otitis Media)		
Mucosal	44	66.6%
Squamous	22	33.3%

Types of deviated nasal septum

In this table, the maximum number of patients were observed to have type 6 deviated nasal septum (DNS), which was 31.81%, and the least were type 7, which was 6.06%. Also, amongst the patients enrolled for this study, none had type 1, 2, 9, or 10 DNS. The rest of the details are explained in Table 2. 

**Table 2:** Distribution of patients as per Jain Bhalerao anatomic endoscopic classification of deviated nasal septum DNS: Deviated nasal septum

TYPE OF DNS	No of Patients (n=66)
Type 3	11 (16.6%)
Type 4	08 (12.12%)
Type 5	17 (25.75%)
Type 6	21 (31.81%)
Type 7	04 (6.06%)
Type 8	05 (7.57%)

Difference between preoperative and postoperative Eustachian tube dysfunction in various types of deviated nasal septum

Table 3 displays the preoperative and postoperative Eustachian tube dysfunction in various types of deviated nasal septum. There was 100% improvement in patients with Eustachian tube dysfunction from grade 2A to 1A in patients with type 3 and type 4 DNS having cartilaginous deviations. In type 5, it showed roughly 94.1% improvement, where out of 16 patients having a 3A grade and one patient with a 2A grade, five (7.5%) patients and 11 (16.6%) patients improved to grades 2A and 1A, respectively, while one (1.5%) patient showed no improvement. In type 6 bony deviations, all 21 patients showed improvement, of which 11 (16.6%) patients and 10 (15.1%) patients improved from grade 3A to 1A and 2A, respectively. In type 7, there was a 50% improvement where two (3.0%) out of four patients showed improvement from grade 3A to 2A, whereas two (3.0%) patients showed no improvement. In type 8, 80% of patients showed improvement, where two (3.0%) out of five patients improved from grade 3A to 2A, two (3.0%) patients improved from grade 3A to 1A, and one (1.5%) patient showed no improvement. In this graph, the association in type 5, type 6, and type 8 is of statistical significance with p-values < 0.001, < 0.001, and 0.0357, respectively.

**Table 3:** Comparison of difference between pre-operative and post-operative values with improvement in grade of Eustachian tube function DNS: Deviated nasal septum, ETD: Eustachian tube dysfunction, Chi2: Chi-square, Pre-op: Pre-operative, Post-op: Post-operative. Eustachian tube function: Grades zero and 1A were considered normal functions; 2A: mechanical dysfunction, edema, and congestion at the lumen; the lumen opens partly on swallowing; 3A: mechanical dysfunction; the tubal lumen fails to open with swallowing due to gross edema. In this table, a chi-square test was used, and the association in types 5, 6, and 8 was of statistical significance with p-values < 0.001, < 0.001, and 0.0357, respectively.

Type of DNS	Pre-op ETD (n=66)			Post-op ETD (n=66)			% Improvement	Chi sq	p-value
	0/1A	2A	3A	0/1A	2A	3A			
Type 3	-	11 (16.6%)	-	11(16.6%)	-	-	100%	NA	-
Type 4	-	08 (12.1%)	-	08 (12.1%)	-	-	100%	NA	-
Type 5	-	01 (1.5%)	16 (24.2%)	11 (16.6%)	05 (7.5%)	01 (1.5%)	94.1%	Chi2: 26.902	p < 0.001
Type 6	-	-	21 (31.8%)	11 (16.6%)	10 (15.1%)	-	100%	Chi2: 42.0	p-value <0.001
Type 7	-	-	04 (6.0%)	-	02 (3.0%)	02 (3.0%)	50%	Chi2: 2.66	p-value < 0.1025
Type 8	-	-	05 (7.5%)	02 (3.0%)	02 (3.0%)	01 (1.5%)	80%	Chi2: 6.6667	p-value < 0.0357

Comparative assessment of nasal resistance on the side of DNS (pre- and post-septoplasty)

In the preoperative state, types 3 and 4 had 100% moderate nasal resistance. Type 5 had a mix of moderate (23.5%) and severe (76.4%) nasal resistance. Types 6, 7, and 8 had 100% severe nasal resistance. Post-operative state: types 3 and 4 improved to 100% mild cases. Types 5, 6, 7, and 8 showed significant improvements, with a shift towards mild and moderate cases and no severe cases remaining. This visualization clearly shows the positive impact of the surgical intervention that is septoplasty across all DNS types, with a notable reduction in severe nasal resistance as depicted in Table 4.

**Table 4:** Comparison of the difference between pre-operative and post-operative changes in nasal resistance and its improvement post-septoplasty Nasal resistance: Normal value: 0.15 to 0.36 Pa/cm3/sec, Mild: 0.36 to 0.47 Pa/cm3/sec, Moderate: 0.48 to 0.59 Pa/cm3/sec, Severe: > 0.59 Pa/cm3/sec [18]. A chi-square test was used for the analysis. This association of nasal resistance pre-operatively and postoperatively in types 7 and 8 is of statistical significance, with p-values of 0.0183 and 0.0067, respectively.

Types of Deviated nasal septum (DNS)	Pre-op nasal resistance (n=66)			Post-op nasal resistance (n=66)			Chi-square test	p-value
(n)	mild	Mod	severe	mild	mod	severe		
Type 3 (n=11)	-	11 (100%)	-	11 (100%)	-	-	-	-
Type 4 (n=8)	-	8 (100%)	-	8 (100%)	-	-	-	-
Type 5 (n=17)	-	4 (23.5%)	13 (76.4%)	10 (58.8%)	7 (41.1%)	-	Chi2 – 23.8182	p-value < 0.001
Type 6 (n=21)	-	-	21 (100%)	9 (42.8%)	12 (57.1%)	-	Chi2- 42.0	p-value <0.001
Type 7 (n=4)	-	-	4 (100%)	3 (75%)	1 (25%)	-	Chi2- 8.0	p-value - 0.0183
Type 8 (n=5)	-	-	5 (100%)	3 (60%)	2 (40%)	-	Chi2- 10.0	p-value - 0.0067

## Discussion

In this study, there was a definite correlation between Eustachian tube dysfunction, assessed through dynamic slow-motion video endoscopy (DSVE), and nasal resistance measured using active anterior rhinomanometry (rhinodebitometry), in patients diagnosed with chronic otitis media with deviated nasal septum, to have an objective parameter of assessment for the contribution of different types of DNS in the causation of Eustachian tube dysfunction (ETD). The present study was undertaken to validate the Jain Bhalerao Classification of DNS as a standard tool for identifying the association of different types of DNS with varying degrees of Eustachian dysfunction [11].

In our study, the age range encompassed a diverse spectrum, with the youngest patient being 18 and the eldest patient being 66 years. The ratio of males to females was calculated to be 1.8:1, indicating a higher representation of the male gender in our study. A similar study of the impact of nasal obstruction on Eustachian tube function- correlation between rhinomanometric and tubal manometric measurements conducted by Enache R et al. included a study population of 139 patients. Of these 68 were males and 71 were females, with a male-to-female ratio of 1:1.04 [15].

In our study involving 66 patients, all participants were diagnosed with chronic otitis media (COM) and deviated nasal septum (DNS) with Eustachian tube dysfunction (ETD). The aural examination findings included pars tensa and pars flaccida retractions and perforation, and accordingly, the middle ear pathology was categorized as mucosal or squamous. The chief nasal symptoms comprised nasal obstruction and snoring. In a similar study conducted by Seibert et al., the authors described the typical presentation of Eustachian tube dysfunction (ETD) patients, who often report a constellation of symptoms such as aural fullness or clogging, pain or discomfort, hearing impairment, tinnitus, and vertigo [1]. The findings from our study and the observations by Seibert et al. highlight the diverse array of symptoms associated with ETD and COM.

In our study, the maximum number of patients (31.8%) had type 6 or high posterior DNS with contralateral bony deviation with maxillary deviation and bony spur, followed by 25.7% in type 5 with high posterior deviation with ipsilateral bony deviation with bony spur. In a similar study by Jain et al., our results align with their work; type 6 DNS was also the most prevalent, occurring in 23% of cases, while type 5 was the second most common, seen in 11.3% of patients [11].

Our findings revealed that 20 patients (30.3%) exhibited Grade 2A ETD on the affected side, characterized by moderate impairment of tubal function. Furthermore, a significant proportion of 46 patients (69.6%) demonstrated Grade 3A ETD on the affected side, indicating severe Eustachian tube dysfunction. In a similar study by Mathew et al., 61 Eustachian tubes were studied. Thirty-seven Eustachian tubes were found to be normal, whereas 24 Eustachian tubes were dysfunctional, of which 10 were patulous type [12]. DSVE is a very useful tool for the study of Eustachian tube dysfunction, as it classifies the dysfunction as mechanical or functional. Also, it grades the dysfunction based on the edema of the torus tubaris and opening of the Eustachian tube with swallowing, which is a physiological method of assessment [16]. At the same sitting, the diagnostic nasal endoscopy will also delineate the likely cause of ETD. The grading system employed in our study classified Grade 0 and Grade 1A as indicative of normal Eustachian tube function, while Grade 2A and Grade 3A were considered abnormal, reflecting varying degrees of tubal dysfunction. We also emphasize the need for standardization of the method for diagnosing Eustachian dysfunction, emphasizing DSVE, which helps not only to identify type but also grade of ETD under direct visualization and identify other nasal, paranasal, and nasopharyngeal pathologies in the same sitting.

In our study, 16.6% of patients with Type 3 DNS and 12.1% of patients with Type 4 DNS exhibited a complete resolution of ETD, with improvement from Grade 2A to Grade 1A, representing a 100% improvement rate. In the case of Type 5 DNS, a substantial improvement of 94.1% was observed. Among the 16 patients with type 6 DNS who had preoperative grade 3A and 1 patient with grade 2A ETD, five patients (7.5%) improved to grade 2A, and 11 patients (16.6%) improved to grade 1A postoperatively. However, one patient (1.5%) did not exhibit any improvement. It was observed that high posterior bony deviations, mainly type 6, 7, and 8, having bony spurs, and maxillary deviation were associated with severe grades of ETD, possibly due to altered nasal cycle causing stasis of fluids and edema of the tube, which improved to mild to moderate ETD after septoplasty. A similar study conducted by Son S et al. investigated the effects of septoplasty on eustachian tube dysfunction (ETD) and its correlation with nasal septal deviation (NSD). The researchers evaluated the prevalence of ETD before and after surgical intervention, with a particular emphasis on the laterality of NSD [19]. The study employed a meta-analytic approach and found that the incidence of ETD was significantly higher in the preoperative period compared to the postoperative period, approximately 4.46 times higher before septoplasty than one month after the procedure. Furthermore, preoperative ETD was observed to be substantially more prevalent on the deviated side of the nasal septum, with a fourfold higher incidence compared to the non-deviated side [19]. The analysis confirmed that in cases of NSD, the likelihood of impaired Eustachian tube function was approximately twice as high on the deviated side when compared to the non-deviated side. This finding was corroborated by tympanometric assessments, which revealed significant differences in the tympanogram results before and after septal surgery, specifically on the deviated side.

We observed that post-septoplasty, the volume of the affected side of the nasal cavity increased, thus reducing the negative pressure and improving the clearance of nasal fluids and, hence, the Eustachian tube function. We also emphasize ruling out sinonasal and nasopharyngeal pathology, likely causing aural symptoms. We also suggest considering septoplasty as the initial management in patients with chronic otitis media with severe Eustachian tube dysfunction with deviated nasal septum, as observed in our study.

In our study, we assessed nasal resistance using active anterior rhinomanometry in 66 patients, both preoperatively and postoperatively, to evaluate the impact of surgical intervention on nasal patency. The findings revealed a significant improvement in nasal resistance across various types of nasal septal deformities (DNS). Due to compliant nasal walls in the cartilaginous region observed in type 3 and type 4 of the Jain Bhalerao classification, exhibiting moderate nasal resistance on the affected side preoperatively showed 100% improvement was observed postoperatively. In type 5 DNS, 23.5% had moderate nasal resistance, and 76.4% had severe nasal resistance preoperatively; 58.8% of patients improved to mild nasal resistance, while 41.1% showed a reduction to moderate nasal resistance, with a statistically significant p-value < 0.001. 21 patients with type 6 DNS exhibited severe nasal resistance on the affected side preoperatively which improved to mild nasal resistance in 42.8% of patients, and 57.1% improved to moderate nasal resistance, with a statistically significant p-value < 0.001. These findings are consistent with a similar study by Kaneda et al., which concluded that endoscopic sinus surgery (ESS) along with septoplasty significantly improves nasal resistance, a key factor strongly correlated with nasal obstruction, a primary symptom of chronic rhinosinusitis [20]. The improvement in nasal resistance was attributed to direct changes resulting from surgical intervention and indirect changes, such as alleviation of paranasal sinus disease and reduction in mucosal edema. Postoperatively, the nasal resistance was found to be 63.4 in bilateral patients and 44.9 in the affected side of unilateral patients, indicating a substantial improvement [20].

In our study, we observed that patients with high posterior bony DNS with maxillary deviation with bony spur were found to have severe nasal resistance, possibly indicating a non-compliant fixed wall of the nasal septum leading to smaller nasal cavity volume on the affected side, causing negative pressure as per Bernoulli’s principle and hence the increased resistance. Conversely, in patients with cartilaginous deviation of the nasal septum, a flexible, pliable wall could alter the volume of the nasal cavity; hence, the nasal resistance was found to be moderate on the affected side. The reduced diameter on the affected side of DNS was observed to likely cause an increase in the airflow velocity at the region of deviation and reduced pressure as per Hagen Poiseuille’s law [15,17]. This pressure drop most likely causes an increase in nasal resistance on the affected side, causing edema of the tubal end and hence resulting in Eustachian dysfunction [15].

We observed that there was a positive correlation in patients with severe DNS that was associated with higher grades of Eustachian dysfunction and severe nasal resistance. This is probably the first study to have correlated the effect of different types of deviated nasal septum with eustachian tube dysfunction and nasal resistance in patients with chronic otitis media using an objective DNS classification system, the Eustachian function assessment tool, and nasal resistance assessment.

Limitations

The limitations of this study include small sample size, the inability to localize the exact region with maximum nasal resistance using active anterior rhinomanometry (rhinodebitometry), and no studies for comparison as it is a newly introduced method of rhinomanometry. Lack of long-term follow-up of the patients.

Strengths

This is probably the first study to have correlated the effect of different types of deviated nasal septum with eustachian tube dysfunction and nasal resistance in patients with chronic otitis media using an objective DNS classification system, the Eustachian function assessment tool, and nasal resistance assessment. This study recommends the necessity to assess the eustachian tube dysfunction and classify the deviated nasal septum in patients with chronic otitis media as per endoscopic anatomic classification and has laid down the guidelines for consideration of septal correction surgery prior to tympano-mastoid surgery. The study introduces an objective test, active anterior rhinomanometry (rhinodebitometry), that could prove valuable for clinicians in objectively evaluating nasal function and guide clinicians in making informed decisions regarding the management of nasal conditions that impact nasal airflow and resistance, monitoring the effectiveness of chosen therapies, and evaluating the improvement or resolution of nasal resistance after treatment.

Areas of further research

Comparative studies with different types of rhinomanometry and a larger sample size. Cohort studies to observe the impact of septal correction surgeries on the reversal of Eustachian dysfunction and squamous chronic otitis media. Studies comparing nasal volume in patients with DNS with rhinomanometry findings.

## Conclusions

Our study validates the role of the utility of septoplasty in the correction of nasal septal deviation and Eustachian tube dysfunction in patients with chronic otitis media. Septoplasty helped in improving the volume of the nasal cavity, thus improving airflow velocity. This helped increase nasal pressure and nasal clearance, thus reducing nasal resistance and improving ETD. In types of DNS with bony deviations, mainly types 6, 7, and 8, the correction of the luminal discrepancy improved ipsilateral nasal resistance. However, Eustachian tube function was improved on both sides. Our study concludes improvement in bony discrepancy improves Eustachian tube function. This is confirmed by clinical observation, improvement in symptoms of the patients like ear discharge, reduced hearing, reversal of active COM to inactive type, and reduction in recurrence of the symptoms. Septoplasty prevents the progression of inactive to active COM, provides better graft uptake, and improves surgical outcomes in surgeries for COM like tympanoplasty and tympano-mastoid exploration. It prevents the recurrence of COM and provides long-term better hearing outcomes.

## References

[1] Seibert JW, Danner CJ (2006). Eustachian tube function and the middle ear. Otolaryngol Clin North Am.

[2] Bluestone CD, Cantekin EI, Beery QC, Stool SE (1978). Function of the Eustachian tube related to surgical management of acquired aural cholesteatoma in children. Laryngoscope.

[3] Lindeman P, Holmquist J (1987). Mastoid volume and eustachian tube function in ears with cholesteatoma. Am J Otol.

[4] Casale J, Hatcher JD (2021). Physiology, Eustachian Tube Function. https://europepmc.org/article/nbk/nbk532284#impact.

[5] Helliwell T (2010). Inflammatory diseases of the nasal cavities and paranasal sinuses. Diagn Histopathol (Oxf).

[6] Souza CD, Bhaya MH, Wagh SP The role of nasal and sinus surgery in otitis media. Oper Techn Otol-Hea Nec Sur.

[7] Akazawa K, Doi H, Ohta S (2018). Relationship between Eustachian tube dysfunction and otitis media with effusion in radiotherapy patients. J Laryngol Otol.

[8] Akyildiz MY, Özmen ÖA, Demir UL, Kasapoğlu F, Coşkun HH, Basut OI, Siğirli D (2017). Impact of septoplasty on Eustachian tube functions. J Craniofac Surg.

[9] Herrera M, Eisenberg G, Plaza G (2019). Clinical assessment of Eustachian tube dysfunction through the Eustachian tube dysfunction questionnaire (ETDQ-7) and tubomanometry. Acta Otorrinolaringol Esp (Engl Ed).

[10] Smith ME, Takwoingi Y, Deeks J (2018). Eustachian tube dysfunction: A diagnostic accuracy study and proposed diagnostic pathway. PLoS One.

[11] Jain S, Bhalerao P, Singh C A new endoscopic and anatomical classification of deviated nasal septum with clinical relevance. Med Sci.

[12] Mathew GA, Kuruvilla G, Job A (2007). Dynamic slow motion video endoscopy in eustachian tube assessment. Am J Otolaryngol.

[13] Szucs E, Clement PA (1998). Acoustic rhinometry and rhinomanometry in the evaluation of nasal patency of patients with nasal septal deviation. Amer J Rhinology.

[14] Choi H, Park IH, Yoon HG, Lee HM (2011). Diagnostic accuracy evaluation of nasal sound spectral analysis compared with peak nasal inspiratory flow in nasal septal deviation. Am J Rhinol Allergy.

[15] Enache R, Sarafoleanu D, Negrila A The impact of nasal obstruction upon Eustachian tube function - a correlation between rhinomanometric and tubal manometric measurements. Romanian Journal of Rhinology, Vol.

[16] Patil N, Jain S, Wadhwa S (2024). Unveiling the potential: a comprehensive review of dynamic slow-motion video endoscopy for Eustachian tube dysfunction evaluation. Cureus.

[17] Patil N, Jain S (2024). Rhinomanometry: a comprehensive review of its applications and advancements in rhinology practice. Cureus.

[18] Kaneda S, Sekine M, Saito K (2019). Evaluation of nasal resistance by rhinomanometry before and after endoscopic sinus surgery. Inter Jr Prac Otola.

[19] Son SP, Youn C, Jin-Hee P, So KY. Comparison of Eustachian tube function before and after septoplasty: a systematic review and meta-analysis. Kor Jr Otor-He Ne Sur.

[20] Corda JV, Shenoy BS, Lewis L, Prakashini K, Khader SMA, Arifin KA, Zuber M (2022810338920221009640). Nasal airflow patterns in a patient with septal deviation and comparison with a healthy nasal cavity using computational fluid dynamics. Front Mech Eng.

